# Effect of extremely low-concentration gaseous chlorine dioxide against surface *Escherichia coli*, *Pseudomonas aeruginosa* and *Acinetobacter baumannii* in wet conditions on glass dishes

**DOI:** 10.1186/s13104-020-4925-5

**Published:** 2020-02-12

**Authors:** Hirofumi Morino, Masafumi Futatsukame, Takanori Miura, Takashi Shibata

**Affiliations:** 1 Taiko Pharmaceutical Co., Ltd., Uchihonmachi 3-34-14, Suita, Osaka 564-0032 Japan; 2 Kyoto Plant/R&D Center, 1-2-1 Hikaridai, Seikacho, Soraku-gun, Kyoto 619-0237 Japan

**Keywords:** Chlorine dioxide, Gas, Bacteria, *Escherichia coli*, *Pseudomonas aeruginosa*, *Acinetobacter baumannii*

## Abstract

**Objective:**

Healthcare-associated infections due to Gram-negative bacteria (GNB) are a major cause of mortality and morbidity throughout the world. The purpose of the research described here was to evaluate the possibility of using an extremely low-concentration gaseous chlorine dioxide (ClO_2_, 0.01 ppmv, 0.028 mg/m^3^) as a technique to reduce the risk of environmental infection by GNB. In this study we set up an exposure chamber (1 m^3^) and used three types of GNB, namely *Escherichia coli*, *Pseudomonas aeruginosa* and *Acinetobacter baumannii*.

**Results:**

The extremely low-concentration gaseous ClO_2_ inactivated *E. coli* (> 2 log_10_ reductions, within 2 h), *P. aeruginosa* (> 4 log_10_ reductions, within 2 h) and *A*. *baumannii* (> 2 log_10_ reductions, within 3 h) in wet conditions on glass dishes. Treatment of moist environments with extremely low-concentration gaseous ClO_2_ may help to reduce the risk of environmental infection by GNB without harmful effects.

## Introduction

Healthcare-associated infections (HAIs) due to Gram-negative bacteria (GNB) are a major cause of mortality and morbidity throughout the world [1]. Many GNB cause respiratory tract infections; these include *Pseudomonas aeruginosa*, *Escherichia coli, Acinetobacter baumannii* and *Klebsiella pneumonia* [2]. In a previous study, we showed that gaseous chlorine dioxide (ClO_2_, CAS No. 10049-04-4) in a low concentration (mean 0.05 ppmv, 0.14 mg/m^3^) inactivates *E. coli* in wet conditions on a glass surface [3]. Recently, Ogata et al. reported that extremely low-concentration gaseous ClO_2_ (0.01 ppmv, 0.028 mg/m^3^) inactivated airborne bacteria and viruses [4]. This concentration is only 1/10 of the threshold limit value (0.1 ppmv, 0.28 mg/m^3^) for gaseous ClO_2_ defined by the American Occupational Safety and Health Administration (OSHA) as an 8 h time-weighted average [5]. Based on that report, we speculated that extremely low-concentration gaseous ClO_2_ can also inactivate bacteria on surfaces. To investigate the efficacy of extremely low-concentration gaseous ClO_2_ against surface bacteria, we set up an exposure chamber (1 m^3^), which can maintain a constant, extremely low- concentration of the gas. In this study, we used three types of bacterium, namely *E. coli*, *P. aeruginosa* and *A. baumannii*. The purpose of this study is to evaluate the usefulness of this treatment with extremely low-concentration gaseous ClO_2_ as a technique to reduce the risk of infection by environmental GNB without harmful effects.

## Main text

### Materials and methods

#### Test bacteria and preparation

Preparation of the test bacteria followed our previous report with some modifications [3]. *E. coli* NBRC 3972, *P. aeruginosa* NBRC 13275 and *A. baumannii* NBRC 110494 were obtained from the Biological Resource Center (NITE, Japan). Each bead from the stock vials (PL.170/M, Microbank™, Pro-Lab Diagnostics Inc., U.S.A.) of *E. coli* and *P. aeruginosa* stored at − 80 °C was transferred into 5 ml of Soybean Casein Digest (SCD) broth (393-00185, Nihon Seiyaku, Japan). Incubation was carried out in a shaking incubator at 200 rpm for 18 h at 37 °C. One bead from a stock vial of *A. baumannii* was transferred onto a SCD agar plate (51048, Nissui Pharmaceutical Co., LTD., Japan) and incubated at 37 °C for 18 h. Cells of *E. coli* and *P. aeruginosa* were collected by centrifugation at 1400×*g* for 15 min at room temperature. Cells of *A. baumannii* on the agar plate were collected with an inoculating loop. These bacteria were washed three times with Dulbecco’s phosphate buffer saline (D-PBS). The bacteria were resuspended in D-PBS and adjusted to an OD_660_ of 0.1 (1 × 10^8^ cells/ml). These bacteria were used for inoculation.

A 100 µl aliquot of the bacterial suspension (1 × 10^8^ cells/ml in D-PBS) was placed on 5 cm diameter glass dishes. In this study, bacteria on the glass dishes did not undergo a drying process. The dishes were placed in the 1 m^3^ exposure chamber described below. The preparations were exposed to 0.01, 0.03 ppmv ClO_2_ gas or air (control) for 0, 1, 2 and 3 h. Subsequently, 120 µl of SCD medium with 1 mM Na_2_S_2_O_3_ as neutralizer was added to the glass dishes after the exposure to air or ClO_2_ gas. The bacteria on the glass dishes were collected using a cell scraper (179693, Thermo Fisher Scientific, U.S.A.).

#### Determination of viable counts

The bacteria samples were serially diluted tenfold and 100 µl of diluted bacteria was inoculated on SCD agar. The bacteria were cultured at 35 °C for 24 to 48 h and the number of colonies on SCD agar was counted. The viable cell counts were determined as colony forming units (CFU)/dish.

#### Exposure chamber

**Fig. 1:**
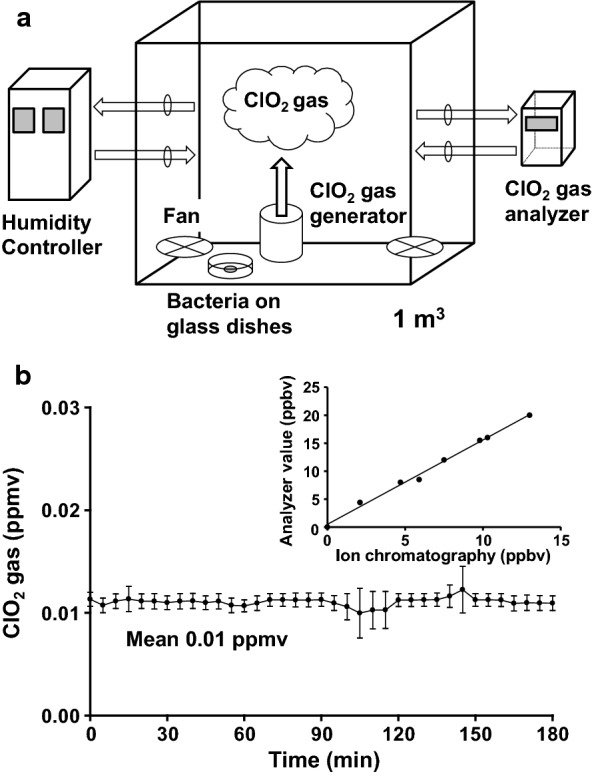
Illustration of the exposure chamber setup and changes in the concentration of gaseous ClO_2_ in the exposure chamber. **a** Schematic diagram of bacteria on glass dishes in an exposure chamber. The bacteria were placed in 1 m^3^ exposure chamber in wet conditions on glass dishes. **b** Changes in the concentration of gaseous ClO_2_ in the exposure chamber. The error bar in the graph indicates S.D. (*n* = 4). The inset shows correlation between gaseous ClO_2_ concentrations (ppbv) by ion chromatography versus measurements using ClO_2_ gas analyzer

We prepared a cubic exposure chamber (1 m^3^) to evaluate the bactericidal effect of the extremely low concentration gaseous ClO_2_ (Fig. 1a). The ClO_2_ gas was introduced into the exposure chamber by a ClO_2_ gas generator which was made in our laboratory. The humidity in the exposure chamber was constantly controlled by a humidity controller (ADPAC-N1000-AH, ADTEC Corporation, Japan). To maintain a homogeneous concentration of ClO_2_ gas in the exposure chamber, the contained air was constantly circulated using two fans (MU1225S-11, ORIX, Japan). The glass dishes inoculated with bacteria were placed in the exposure chamber. The concentration of gaseous ClO_2_ in the exposure chamber was measured by a ClO_2_ gas analyzer (Midas Gas Detector, MIDAS-E-BR2, Honeywell Analytics, IL, U.S.A.). Furthermore, the determination of concentration of ClO_2_ gas was performed using an established protocol (OSHA Method ID-202) with some modifications. Briefly, the ClO_2_ gas was collected in a midget impinger (1448-02, SOGORIKAGAKU GLASS WORKS, Japan) containing 20 ml of 1.2 mM potassium iodide (KI) in a 1.5 mM Na_2_CO_3_/1.5 mM NaHCO_3_ buffer solution at 0.5 L/min for 20 min. The collected ClO_2_ (as ClO_2_^−^) was analyzed by an ion chromatograph (ICS-3000, Thermo Fisher Scientific (DIONEX), U.S.A.). The temperature and relative humidity in the exposure chamber were measured by a thermo-hygrometer (TR-72 wf, T&D, Japan; accuracy of ± 0.3 °C and ± 2.5%RH).

#### Statistical analysis

The presence of any significant difference was determined by Student’s *t* test (two-tailed).

### Results

#### Determination of gaseous ClO_2_ concentration in the exposure chamber

The concentration of gaseous ClO_2_ measured by the gas analyzer in the exposure chamber is shown in Fig. 1b. As shown in Fig. 1b (inset), there was a linear correlation between gaseous ClO_2_ concentrations determined by ion chromatography and measurements by the ClO_2_ gas analyzer; the line was then fitted to a linear function by regression analysis. The values obtained by the ClO_2_ gas analyzer were corrected using the regression equation. The coefficient of determination R^2^ of this fitting was 0.994. The average value of concentration of gaseous ClO_2_ was 0.01 ± 0.001 ppmv. The temperature and relative humidity in the exposure chamber were 24.4 ± 0.2 °C and 56.6 ± 1%, respectively.

#### Bactericidal activity of gaseous ClO_2_ at extremely low concentrations

**Fig. 2:**
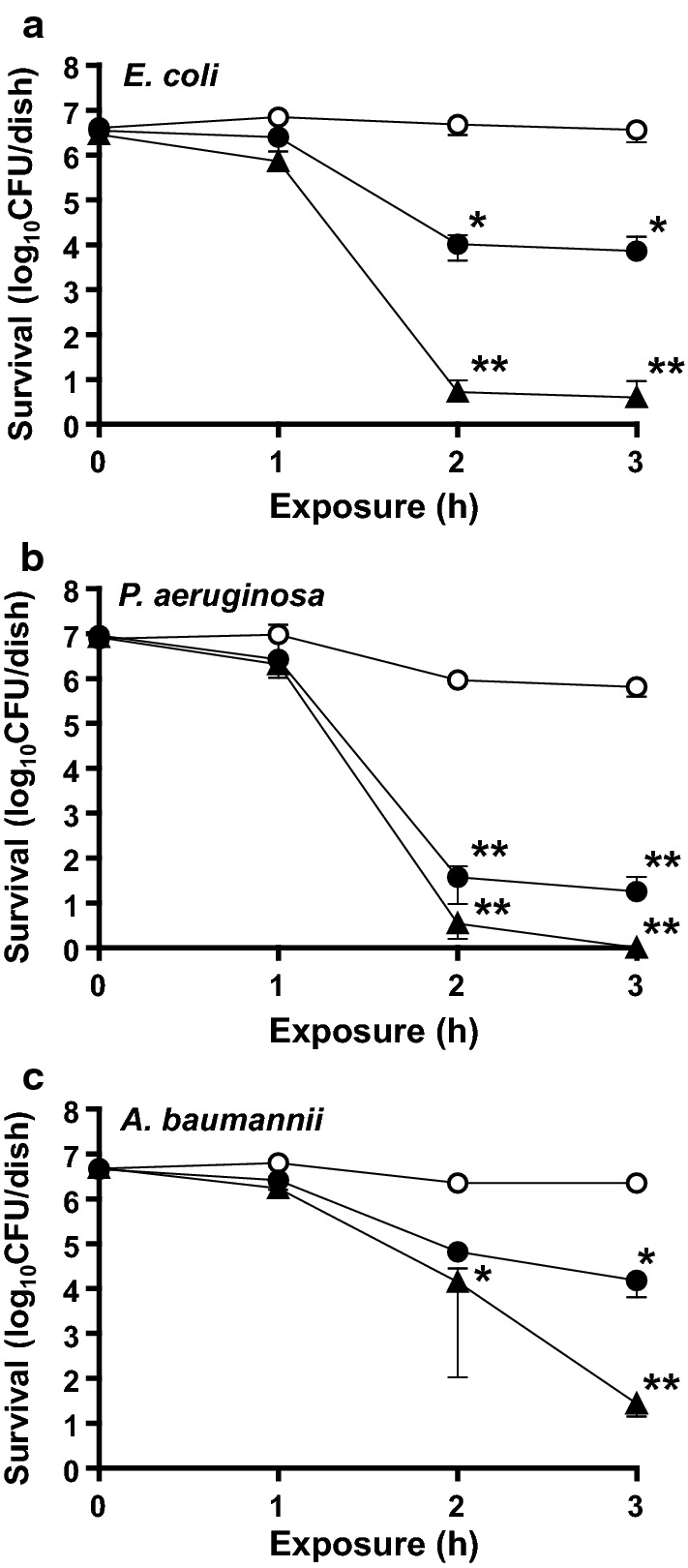
Inactivation of *E. coli* (**a**), *P. aeruginosa* (**b**) and *A. baumannii* (**c**) in wet conditions by extremely low concentration gaseous ClO_2_. The asterisks show reductions of > 2 log_10_ (*) and > 4 log_10_ (**) as compared to control values. Open circle, air; filled circle, 0.01 ppmv ClO_2_ gas; filled triangle, 0.03 ppmv ClO_2_ gas. The bacteria on the glass dishes did not undergo a drying process. No organic matter was mixed with test bacteria in these experiments. Data represent mean ± S.D. (*n* = 4)

We evaluated the bactericidal activity of gaseous ClO_2_ at extremely low-concentrations against *E. coli*, *P. aeruginosa* and *A. baumannii* in wet conditions on glass dishes. The bactericidal activity of gaseous ClO_2_ against *E. coli* exhibited reductions of > 2 log_10_ (mean 0.01 ppmv, *p* < 0.05) and > 4 log_10_ (mean 0.03 ppmv, *p* < 0.05) after 2 h as compared to the control values (Fig. 2a). The bactericidal activity of gaseous ClO_2_ against *P. aeruginosa* exhibited reductions of > 4 log_10_ (mean 0.01 ppmv, *p* < 0.01) after 2 h as compared to the control values and achieved undetectable levels (< 1 CFU/dish) after 3 h (mean 0.03 ppmv, *p* < 0.05) (Fig. 2b). The bactericidal activity of gaseous ClO_2_ against *A. baumannii* showed reductions of > 2 log_10_ (mean 0.01 ppmv, *p* < 0.01) and > 4 log_10_ (mean 0.03 ppmv, *p* < 0.01) after 3 h as compared to the control values (Fig. 2c).

#### Bactericidal activity of gaseous ClO_2_ under dirty conditions

Efficacy of gaseous ClO_2_ against microbes in wet conditions was decreased in the presence of fetal bovine serum (FBS) as an organic substance [3]. Hence, we examined the effect of an FBS load (0, 3, 5 and 10%) on the inactivation of *P. aeruginosa*, which had the highest sensitivity to gaseous ClO_2_ of the three evaluated bacteria, with a ClO_2_ gas of concentration of 0.01 ppmv. The extremely low- concentration gaseous ClO_2_ (0.01 ppmv) inactivated *P. aeruginosa* with 5% FBS (2.9 log_10_ reductions) within 3 h (Table 1).

**Table 1:** Effect of organic substance load on bactericidal activity against *P. aeruginosa* of extremely low concentration gaseous ClO_2_ in wet conditions

Exposure time (h)	FBS concentration in bacterial suspension (%)	*P. aeruginosa* survival (log_10_ CFU/dish)	
		Air	ClO_2_
3	0	6.2	0.1 (6.1^b^)
	3	7.0	3.3 (3.7^a^)
	5	6.9	4.0 (2.9^a^)
	10	6.7	5.2 (1.5)

Values in parentheses in the table indicate log_10_ reduction. Survival values of *P. aeruginosa* indicate reductions of ^a^> 2 log_10_ and ^b^> 4 log_10_ as compared to control values (Air). Data represent mean value (*n* = 4)

### Discussion

Gaseous agents and disinfectant vapors have excellent diffusive characteristics. Such agents can disinfect areas where liquid agents are difficult to use. However, many procedures using formaldehyde, peracetic acid, etc. have disadvantages, for example, skin inflammation and acute toxicity for the respiratory system. According to the OSHA, the threshold limit value for gaseous ClO_2_ is 0.1 ppmv as an 8 h time-weighted average. The extremely low-concentration gaseous ClO_2_ (0.01 ppmv) used to inactivate GNB in this research was only 1/10 of that concentration. In a previous study, we demonstrated that gaseous ClO_2_ at 0.1 ppmv was not toxic when whole bodies of rats were exposed to the gas for 6 months [6]. Furthermore, Ogata et al. reported that the no observed adverse effect level (NOAEL) of gaseous ClO_2_ was 1 ppmv [7]. From these results, we think that use of extremely low- concentration gaseous ClO_2_ (0.01 ppmv) is a feasible method for inactivating GNB in the presence of humans, without adverse effects.

In this study, the bactericidal activity against multidrug-resistant (MDR)-GNB of gaseous ClO_2_ of extremely low-concentration was not determined. However, a ClO_2_ solution of 10 mg/L drastically reduced the number of MDR-*P. aeruginosa* and MDR-*A. baumannii* bacteria within 60 s under conditions of a mixture containing a high concentration of bovine serum albumin and sheep erythrocytes [8]. Furthermore, the effect of inactivation by a ClO_2_ solution of 10 mg/L against drug-sensitive *P. aeruginosa* (1.4 log_10_ reductions, within 15 s) was lower than that against MDR-*P. aeruginosa* (3.6 log_10_ reductions, within 15 s). In other words, MDR-*P. aeruginosa* are more sensitive to ClO_2_ than drug-sensitive *P. aeruginosa*. These data suggest that extremely low-concentration gaseous ClO_2_ may have a bactericidal effect against MDR-GNB.

In a previous study, we discussed the fact that moisture plays an important role in the inactivation of feline calicivirus on glass dishes by < 0.1 ppmv ClO_2_ gas [9]. Hence, in this study we evaluated bactericidal activity against GNB of extremely low-concentration gaseous ClO_2_ in wet conditions, but not in the dry state. Previous studies are not sufficient to clarify the route of infection for GNB in wet environments such as sinks and bathroom. However, several studies suggest that a watery environment as occurs in kitchens, drains, bathrooms, sinks and faucets serves as a reservoir for microorganisms [10, 11]. Barker et al. showed that flushing a toilet produces aerosols containing microbes that are capable of causing surface contamination within the toilet [12]. In this study, gaseous ClO_2_ at an extremely low- concentration inactivated GNB in wet conditions on glass dishes as a model of microbes on surfaces in a wet environment. In addition, the extremely low-concentration gaseous ClO_2_ inactivated *P. aeruginosa* with 5% FBS (2.9 log_10_ reductions) within 3 h. The percent FBS contained in a microbial suspension (minimum 5% FBS) was designed by the United States Environmental Protection Agency (US EPA) for virucidal effectiveness testing using feline calicivirus [13]. It should be noted that the effect of inactivation of *P. aeruginosa* by extremely low-concentration gaseous ClO_2_ was considerably decreased in the presence of 10% FBS. Therefore, its inactivating effect may be limited under dirty conditions (> 5% FBS). In addition, we examined the effect of an erythrocytes load (final 0.3% (w/v) BSA and 0.3% (v/v) erythrocytes) on the inactivation of *P. aeruginosa* by extremely low-concentration gaseous ClO_2_. The extremely low-concentration gaseous ClO_2_ (0.01 ppmv) inactivated *P. aeruginosa* with erythrocytes (1.0 log_10_ reductions) within 3 h. Taken together, treatment of moist environments with extremely low- concentration gaseous ClO_2_ may help to reduce the risk of environmental infection by GNB without harmful effects.

## Limitations

We were not able to determine the bactericidal activity of gaseous ClO_2_ of extremely low-concentration against multidrug-resistant (MDR)-GNB. We were also not able to evaluate the effect of organic substance load on the inactivation of *E. coli* and *A. baumannii b*y extremely low-concentration gaseous ClO_2_. We were also not able to evaluate bactericidal activity of gaseous ClO_2_ at extremely low-concentrations against GNB in environments.

## Data Availability

All data generated or analyzed during this study are included in this published article.

## Abbreviations

GNB: Gram-negative bacteria
ClO_2_: Chlorine dioxide
SCD: Soybean Casein Digest
HAIs: Healthcare-associated infections
OSHA: Occupational Safety and Health Administration
NITE: National Institute of Technology and Evaluation
D-PBS: Dulbecco’s phosphate buffer saline
CFU: Colony forming units
MDR: Multidrug-resistant
OD: Optical Density
BSA: Bovine serum albumin
